# Cholesterol and steroid synthesis pathways may be involved in the inhibition of osteosarcoma cell viability by calcium-sensing receptor antagonism

**DOI:** 10.7717/peerj.20546

**Published:** 2026-01-05

**Authors:** Luchuan Wang, Jianfa Wang, Xinjie Chu, Yao Liu, Yanping Fan, Xunzhong Qi, Jin Guo, Shuqiu Wang

**Affiliations:** 1 Department of Pathophysiology, School of Basic Medicine, Jiamusi University, Jiamusi, China; 2 Department of Orthopedics, School of Clinical Medicine, Jiamusi University, Jiamusi, China; 3 School of Rehabilitation, Jiamusi University, Jiamusi, China; 4 Department of Neurology, School of Clinical Medicine, Jiamusi University, Jiamusi, China

**Keywords:** Osteosarcoma, CaSR antagonist, Transcriptome sequencing, NPS-2143, Cholesterol synthesis

## Abstract

**Background:**

This study examined the effect of calcium-sensing receptor (CaSR) antagonism on human osteosarcoma cells and investigated the underlying molecular mechanisms of this effect through transcriptome sequencing.

**Methods:**

Human osteosarcoma cell lines MG-63 and Saos-2 were treated with different concentrations (0.1–10 µM) of the CaSR antagonist NPS-2143. Cell Counting Kit-8 (CCK-8) assays were used to detect the effect of CaSR antagonism on the viability of the cells. RNA sequencing was performed on cells treated with five µM NPS-2143 for 24 hours, followed by bioinformatic analysis to identify differentially expressed genes and enriched pathways. qRT-PCR was conducted to validate key genes.

**Results:**

CCK-8 assays showed that at low concentrations (0.1 and one µM), NPS-2143 had no significant effect on MG-63 and Saos-2 cell viability. At higher concentrations (five µM and 10 µM), the viability of MG-63 and Saos-2 cells was significantly reduced. Five µM was therefore selected for subsequent experiments. RNA sequencing revealed distinct gene expression profiles in NPS-2143–treated cells compared to controls. A total of 927 differentially expressed genes (DEGs) were identified in Saos-2 cells (378 upregulated, 549 downregulated), and 59 DEGs were identified in MG-63 cells (33 upregulated, 26 downregulated). Reactome and KEGG pathway enrichment analyses indicated significant enrichment of cholesterol and steroid biosynthesis–related pathways. Transcriptome sequencing showed that NPS-2143 modulated the expression of genes in cholesterol and steroid synthesis pathways. Real-time quantitative reverse transcription polymerase chain reaction (qRT-PCR) confirmed that NPS-2143 promoted the expression of the cholesterol and steroid synthesis pathway genes, *CYP51A1*, *DHCR24*, *LSS*, and *MSMO1* in MG-63 and Saos-2 cells.

**Discussion:**

The inhibitory effect of NPS-2143 on MG-63 and Saos-2 osteosarcoma tumor cell viability was confirmed. CaSR antagonism significantly up-regulated genes involved in cholesterol and steroid biosynthesis, including *CYP51A1*, *DHCR24*, *LSS*, and *MSMO1*. These genes encode key enzymes in the cholesterol synthesis pathway, and their upregulation may lead to cholesterol overproduction. This may, in turn, lead to the formation of oxysterols, which are known to induce inflammation and cytotoxicity. These findings suggest a potential metabolic mechanism through which CaSR antagonists influence osteosarcoma cell viability. Although further validation is warranted, our results provide preliminary evidence implicating cholesterol biosynthesis as a mechanistic target in osteosarcoma and underscore the exploratory value of CaSR antagonists as metabolic regulators in cancer research.

## Introduction

Osteosarcoma, the most common primary malignant bone tumor, affects primarily children and adolescents. It is highly invasive and prone to distant metastasis, making it clinically challenging (Mirabello, Troisi & Savage, 2009; Ying et al., 2023). Currently, the standard treatment for osteosarcoma involves surgical resection in combination with neoadjuvant and adjuvant chemotherapy. While this multimodal approach has significantly improved patient survival over the past few decades, recurrence and metastasis remain major obstacles, and the five-year survival rate remains low (Marina et al., 2004; Mehrvar et al., 2023). Therefore, the identification of new therapeutic targets and effective drug regimens is essential for improving patient outcomes.

The calcium-sensing receptor (CaSR) is a G-protein-coupled receptor widely expressed in various tissues that is primarily responsible for regulating calcium ion (Ca^2^^+^) homeostasis (Alqudah et al., 2022; Brown & MacLeod, 2001). CaSR plays a crucial role in the development and progression of multiple malignant tumors, with its activation or inhibition influencing tumor cell proliferation, apoptosis, invasion, and metastasis (Chakravarti et al., 2009; Wolffs et al., 2025). In bone-related diseases, the role of CaSR is particularly significant. CaSR is aberrantly expressed in osteosarcoma cells and may regulate tumor growth and invasiveness through intracellular calcium signaling pathways (Tuffour et al., 2021). However, its precise role in the progression of osteosarcoma and its potential as a therapeutic target are still poorly understood.

Recent studies indicate that calcium signaling, particularly *via* the CaSR, plays an important role in lipid metabolism. CaSR activation regulates intracellular calcium levels through pathways such as Gq/11 and Gi/o, thereby affecting lipid synthesis and inducing the expression of lipogenic genes in adipocytes and hepatocytes (He et al., 2011b). Calcium availability also influences cholesterol biosynthesis by modulating SREBP transcription factors and key enzymes such as β-Hydroxy β-methylglutaryl-CoA (HMG-CoA) reductase (Wang et al., 2017). Disruption of calcium homeostasis may aberrantly activate SREBPs and enhance cholesterol synthesis, whereas calcium-dependent kinases like AMPK can suppress this process (Jeon, 2016). These insights suggest a potential interplay between CaSR-mediated calcium signaling and lipid–cholesterol metabolic regulation. Although direct evidence remains limited, this relationship implies that CaSR may modulate related metabolic pathways and therefore merits further investigation.

Previous studies have shown that CaSR expression is upregulated in osteosarcoma cells, suggesting its involvement in tumor proliferation and invasiveness (Zhao et al., 2020). Although these studies established a connection between CaSR activity and osteosarcoma cell behavior, the functional consequences of CaSR modulation and the underlying molecular mechanisms remain poorly understood. CaSR antagonists, or negative allosteric modulators of the calcium-sensing receptor, have demonstrated therapeutic potential in several diseases by interfering with calcium-dependent signaling pathways (Kim & Wysolmerski, 2016; Yang et al., 2023). Investigating their transcriptomic effects in osteosarcoma may therefore provide new insights into CaSR-mediated regulatory networks and enhance our understanding of the molecular mechanisms driving tumor progression.

This study was designed as an exploratory investigation to evaluate the effects of CaSR antagonism on human osteosarcoma cells and to uncover associated molecular mechanisms using transcriptomic analysis. Rather than assessing therapeutic selectivity or safety, our aim was to gain mechanistic insights into CaSR-regulated pathways and to identify potential targets for future therapeutic development in osteosarcoma.

## Materials and Methods

### Materials

MG-63 (TCHu124) and Saos-2 (TCHu114) cells were purchased from the Cell Bank of the Committee for the Preservation of Traditional Culture at the Chinese Academy of Sciences in China. Penicillin/ streptomycin (E607011), Cell Counting Kit-8 (CCK-8) (E606335-2000), and Dulbeccos Modified Eagle’s Medium (DMEM, E600003-0500) were purchased from ShenGon Biotech, China. Fetal bovine serum (C0232) was obtained from Beyotime, China. CaSR antagonist, NPS-2143 (HY-10007), was obtained from MedchemExpress, Inc. TRIzol (15596026CN) was purchased from Thermo Fisher Scientific, Rockford, IL, USA. PrimeScript RT Master Mix Kit (RR036A) and TB Green Premix Ex Taq™ II (RR820A) were obtained from Takara, Otsu, Japan.

### Cell culture, cell treatments, and cell viability (CCK-8) assay

MG-63 and Saos-2 cells were used between passages three and 10 for all experiments to ensure consistency. MG-63 cells were cultured in DMEM supplemented with 1% penicillin/streptomycin and 10% fetal bovine serum. Saos-2 cells were cultured in DMEM supplemented with 1% penicillin/ streptomycin and 15% fetal bovine serum, at 37 °C in a humidified 5% CO_2_ atmosphere. The cells were divided into control and NPS-2143 groups and grown in 96-well culture plates. Cells in the NPS-2143 group were treated for 24 h with varying concentrations (0.5, 1, 5, and 10 µM) of the CaSR antagonist NPS-2143, which was dissolved in dimethyl sulfoxide (DMSO) and diluted in phosphate buffered saline (PBS). The control group received an equivalent volume of the DMSO/PBS vehicle to ensure consistency across treatment conditions. Cell viability was determined by adding 10 µl CCK-8 reagent to each well and incubating for 3 h. Absorbance at 450 nm was recorded on a microplate reader.

### mRNA sequencing

The cells were divided into control and NPS-2143 groups. There were three samples in each group, and the sequencing depth was 16X. The statistical power of this experimental design, calculated in RNASeqPower is 0.9915. Total RNA was isolated from cells using TRIzol extraction. For cDNA library preparation, mRNA was enriched using oligo (dT)-conjugated magnetic beads and then fragmented into approximately 300 bp fragments. First- and second-strand cDNA synthesis was carried out using random primers and reverse transcriptase. Following end repair and 3′-adenylation, sequencing adapters were ligated to the cDNA fragments. The resulting libraries were purified, PCR-amplified, and quantified, and paired-end sequencing was performed on the Illumina NovaSeq X Plus platform. RNA-seq libraries were prepared using the Illumina^®^ Stranded mRNA Prep, Ligation kit (Illumina, San Diego, CA, USA) according to the manufacturer’s protocol.

### Bioinformatic analysis

In this study, raw reads were filtered using Fastp (version 0.23.4, https://github.com/OpenGene/fastp) to remove sequencing adapters, reads with more than 10% unknown bases (N), and low-quality bases (Phred score < 20) at the 3′ end. Reads shorter than 20 bp after trimming were also discarded. Additionally, base quality distribution and error rate statistics were evaluated to ensure the integrity of the clean data before downstream analysis. Sequence alignment was performed using the Homo sapiens reference genome (GRCh38), which was downloaded from Ensembl (http://asia.ensembl.org/Homo_sapiens/Info/Index). Sequence alignment and quality evaluation, incorporating reference genome mapping, sequencing depth analysis, and chromosomal distribution assessment, were performed with HiSat2 (Version 2.2.1, available at http://ccb.jhu.edu/software/hisat2/index.shtml). Transcript quantification and expression profiling were carried out using RSEM (Version 1.3.3, obtainable from http://deweylab.github.io/RSEM/).

Differential gene expression analysis was performed using the DESeq2 package, with significance thresholds set at P adjust < 0.01 and absolute fold change (FC) ≥ 1. Functional annotation and pathway enrichment analysis were conducted using the Reactome pathway database and the Kyoto Encyclopedia of Genes and Genomes (KEGG) pathway database to investigate the biological significance of identified differentially expressed genes.

### Quantitative real-time polymerase chain reaction

Saos-2 and MG63 cells were inoculated with a density of 2  × 10^5^/ml and cultured with 6-well plates, and RNA was extracted immediately after experimental intervention. Total RNA was isolated from cells using TRIzol reagent. cDNA was synthesized using the PrimeScript RT reagent kit. Gene expression was analyzed by quantitative real-time polymerase chain reaction (qRT-PCR) employing SYBR Premix Ex Taq II (Takara), with *GAPDH* as the normalization control. Relative quantification of target gene expression was determined using the 2^−ΔΔCt^ method. All oligonucleotide primers for qRT-PCR were commercially obtained from Shanghai BioEngineering Company. The minimum information for publication of quantitative real-time PCR experiments is listed in the Supplemental Material.

### Statistical analysis

Statistical analysis was performed using IBM SPSS version 29.0 software. Quantitative variables are expressed as the mean ± standard error of mean (SEM). The differences among the groups larger than three were evaluated using a one-way analysis of variance (ANOVA) followed by a *post-hoc* test to determine the least significant difference. Differences between the two groups were conducted through Student’s *t*-test, with statistical significance defined as a *P*-value less than 0.05.

## Results

### A CaSR antagonist inhibits the viability of human osteosarcoma MG-63 and Saos-2 cells

**Figure 1:**
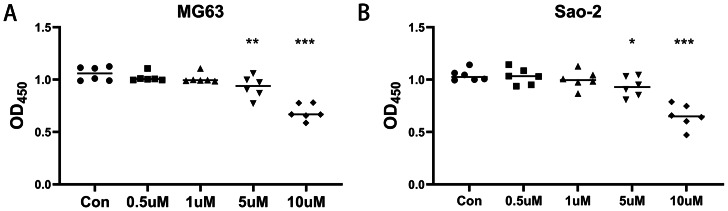
Effect of CaSR antagonism on the viability of human osteosarcoma cells (*n* = 6). (A) MG-63 cells; (B) Saos-2 cells. * *P* < 0.05, ** *P* < 0.01, *** *P* < 0.001.

MG-63 and Saos-2 cells were treated with 0.5, 1, 5, and 10 µM of the CaSR antagonist, NPS-2143, and CCK-8 assays were performed to determine the effect of NPS-2143 on cell viability. There was no significant difference in viability between MG-63 cells treated with 0.1 µM (*P* = 0.386) or one µM (*P* = 0.278) NPS-2143 and the control group. MG-63 cell viability was significantly reduced after treatment with five µM (*P* = 0.005) or 10 µM (*P* = 0.001) NPS-2143 (Fig. 1A). Also, there was no significant difference in cell viability between Saos-2 cells treated with 0.1 µM (*P* = 0.814) or one µM (*P* = 0.423) NPS-2143 and the control group. Saos-2 cell viability was significantly reduced after treatment with five µM (*P* = 0.039) or 10 µM (*P* < 0.001) NPS-2143 (Fig. 1B). Although the reduction in cell viability at five µM was moderate, statistical analysis confirmed that it was significant (*p* < 0.05). This concentration was therefore chosen for transcriptomic analysis, as it enabled the detection of molecular alterations while preserving sufficient cell viability for reliable RNA extraction.

### CaSR antagonism regulates the expression of cholesterol synthesis and steroid synthesis pathway genes in Saos-2 cells

**Figure 2:**
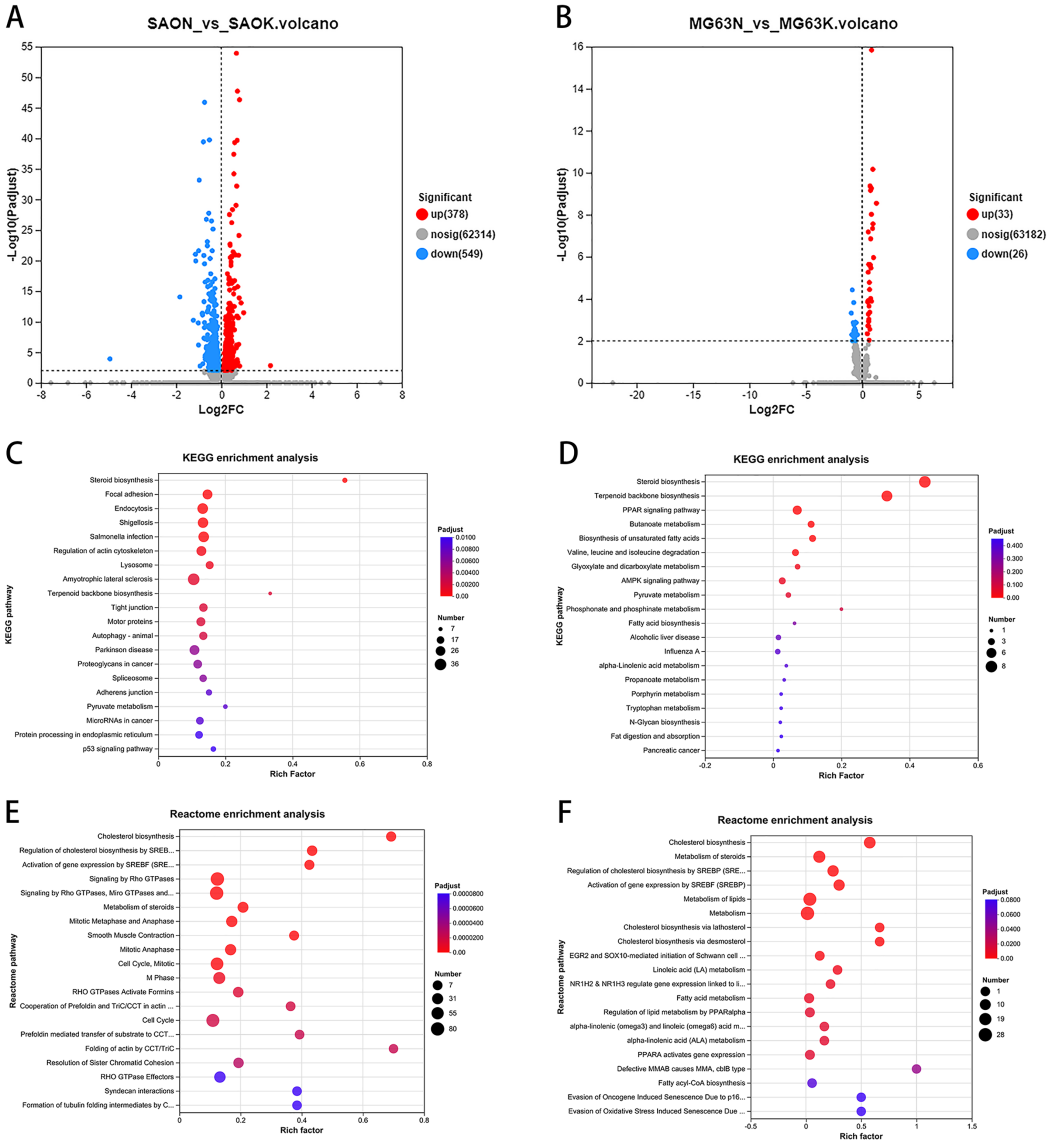
CaSR antagonism regulates the expression of cholesterol synthesis and steroid synthesis pathway genes in Saos-2 and MG-63 cells. (A) Differential gene expression volcano from Saos-2 cells; (B) Differential gene expression volcano from MG-63 cells; (C) KEGG enrichment analysis of Saos-2 cells; (D) KEGG enrichment analysis of MG-63 cells; (E) Reactome enrichment analysis of MG-63 cells; (F) Reactome enrichment analysis of MG-63 cells.

To investigate the effect of a CaSR antagonist on human osteosarcoma cells, Saos-2 cells were treated with five µM NPS-2143 and transcriptome sequencing was performed. We identified 927 differentially expressed genes between the NPS-2143–treated group and the control group, of which 378 genes showed increased expression and 549 genes showed decreased expression (Fig. 2A). KEGG enrichment analysis of the differentially expressed genes showed that the steroid biosynthesis pathway (hsa00100, *P*-adjust < 0.0001, Fig. 2C) had the highest degree of enrichment, the enriched genes including *LIPA (lipase A, lysosomal acid type, LIPA)*, *LBR (lamin B receptor, LBR)*, *HSD17B7 (hydroxysteroid 17-beta dehydrogenase 7, HSD17B7)*, *LSS (lanosterol synthase, LSS)*, *EBP (EBP cholestenol delta-isomerase, EBP)*, *DHCR24 (24-dehydrocholesterol reductase, DHCR24)*, *CYP51A1 (cytochrome P450 family 51 subfamily A member 1, CYP51A1)*, *FDFT1 (farnesyl-diphosphate farnesyltransferase 1, FDFT1)*, *SQLE (squalene epoxidase, SQLE)*, and *MSMO1 (methylsterol monooxygenase 1, MSMO1)*. Reactome enrichment analysis of the differentially expressed genes showed that the cholesterol biosynthesis pathway (R-HSA-191273, *P*-adjust < 0.0001, Fig. 2E) had the highest degree of enrichment, the enriched genes including *FDPS (farnesyl diphosphate synthase, FDPS)*, *LBR*, *SREBF1 (sterol regulatory element binding transcription factor 1, SREBF1)*, *HSD17B7*, *MSMO1*, *SQLE*, *MVD (mevalonate diphosphate decarboxylase, MVD)*, *LSS*, *EBP*, *DHCR24*, *CYP51A1*, *SREBF2(sterol regulatory element binding transcription factor 2*, *SREBF2)*, *FDFT1*, *HMGCR (3-hydroxy-3-methylglutaryl-CoA reductase*, *HMGCR)*, *HMGCS1 (3-hydroxy-3-methylglutaryl-CoA synthase 1*, *HMGCS1)*, *MVK (mevalonate kinase*, *MVK)*, *IDI1 (isopentenyl-diphosphate delta isomerase 1, IDI1)*, and *ACAT2 (acetyl-CoA acetyltransferase 2, ACAT2)*.

### CaSR antagonism regulates the expression of cholesterol synthesis and steroid synthesis pathway genes in MG-63 cells

In MG-63 cells treated with five µM NPS-2143, 59 differentially expressed genes were detected compared with the control group, of which 33 genes showed increased expression and 26 genes showed decreased expression (Fig. 2B). KEGG enrichment analysis of the differentially expressed genes showed that the steroid biosynthesis pathway (hsa00100, *P*-adjust < 0.0001, Fig. 2D) had the highest degree of enrichment, with enriched genes including *NSDHL (NAD(P) dependent steroid dehydrogenase-like, NSDHL)*, *SQLE*, *LSS*, *EBP*, *DHCR24*, *CYP51A1*, *FDFT1* and *MSMO1*. Reactome enrichment analysis of differentially expressed genes showed that the cholesterol biosynthesis pathway (R-HSA-191273, *P*-adjust < 0.0001, Fig. 2F) had the highest degree of enrichment, with enriched genes including *NSDHL (NAD(P) dependent steroid dehydrogenase-like, NSDHL)*, *FDPS (farnesyl diphosphate synthase, FDPS)*, *MSMO1*, *SQLE*, *MVD*, *LSS*, *EBP*, *DHCR24*, *CYP51A1*, *FDFT1*, *HMGCR*, *HMGCS1*, *MVK*, *IDI1*, and *ACAT2*.

### Bioinformatic analysis of common differentially expressed genes between Saos-2 and MG-63 cell lines: CaSR antagonism regulates the expression of cholesterol synthesis and steroid synthesis pathway genes in human osteosarcoma cells

**Figure 3:**
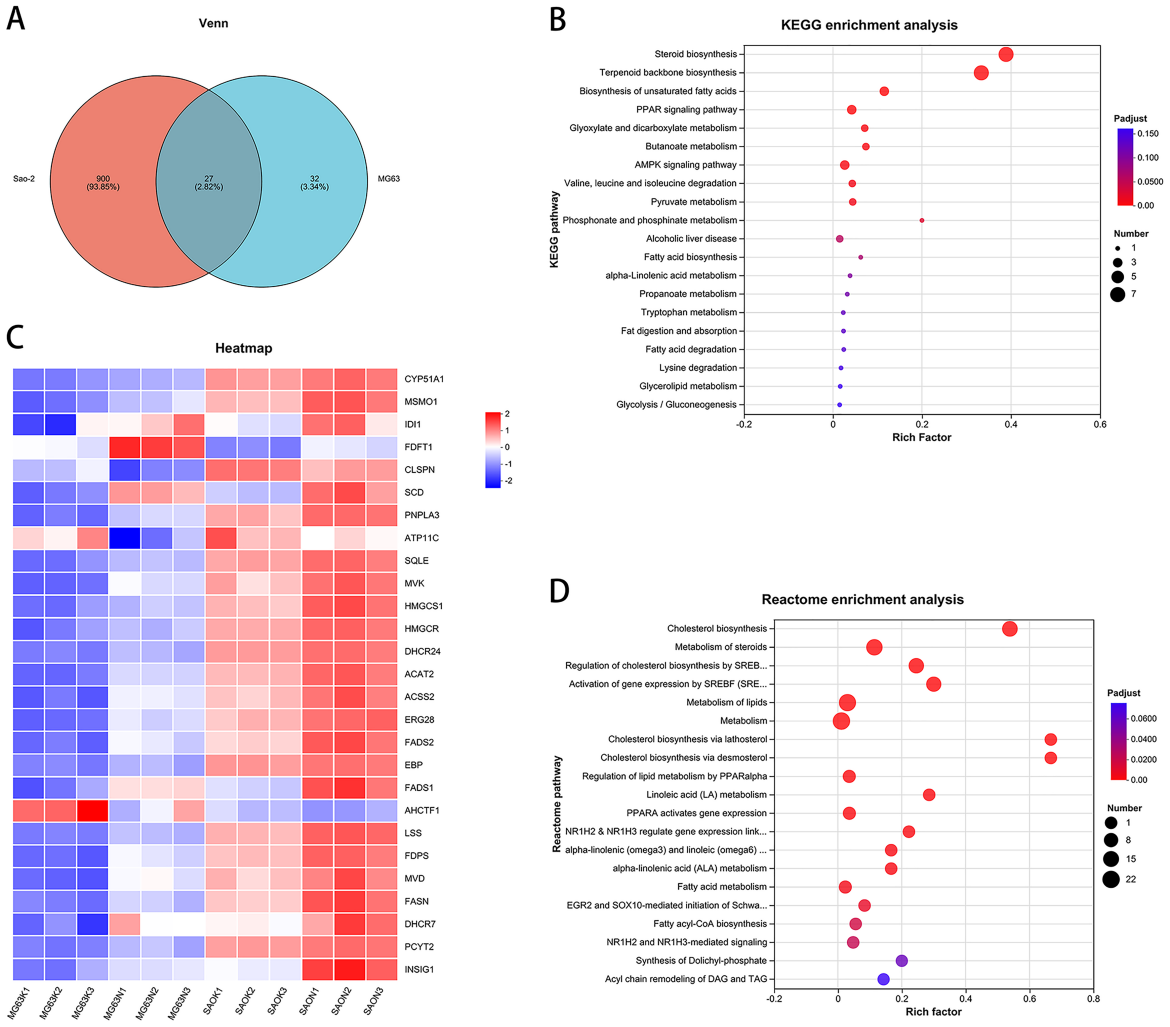
CaSR antagonism regulates the expression of cholesterol synthesis and steroid synthesis pathway genes in human osteosarcoma cells. (A) Venn diagram of differentially expressed genes; (B) KEGG enrichment analysis of human osteosarcoma cells; (C) Differentially expressed gene heatmap; (D) Reactome enrichment analysis of human osteosarcoma cells.

Intersection of the differentially expressed genes from the two cell lines revealed 27 common genes (Figs. 3A and 3C). KEGG enrichment analysis of these genes showed that the steroid biosynthesis pathway (hsa00100, *P*-adjust < 0.0001, Fig. 3B) had the highest degree of enrichment, and that the enriched genes included *SQLE*, *LSS*, *EBP*, *DHCR24*, *CYP51A1*, *FDFT1*, and *MSMO1*. Reactome enrichment analysis of the common differentially expressed genes showed that the cholesterol biosynthesis pathway (R-HSA-191273, *P*-adjust < 0.0001, Fig. 3D) also had the highest degree of enrichment, with enriched genes including *FDPS*, *MSMO1*, *SQLE*, *MVD*, *LSS*, *EBP*, *DHCR24*, *CYP51A1*, *FDFT1*, *HMGCR*, *HMGCS1*, *MVK*, *IDI1*, and *ACAT2*.

### CaSR antagonism promotes expression of genes for cholesterol synthesis and steroid synthesis pathways in human osteosarcoma cells

**Figure 4:**
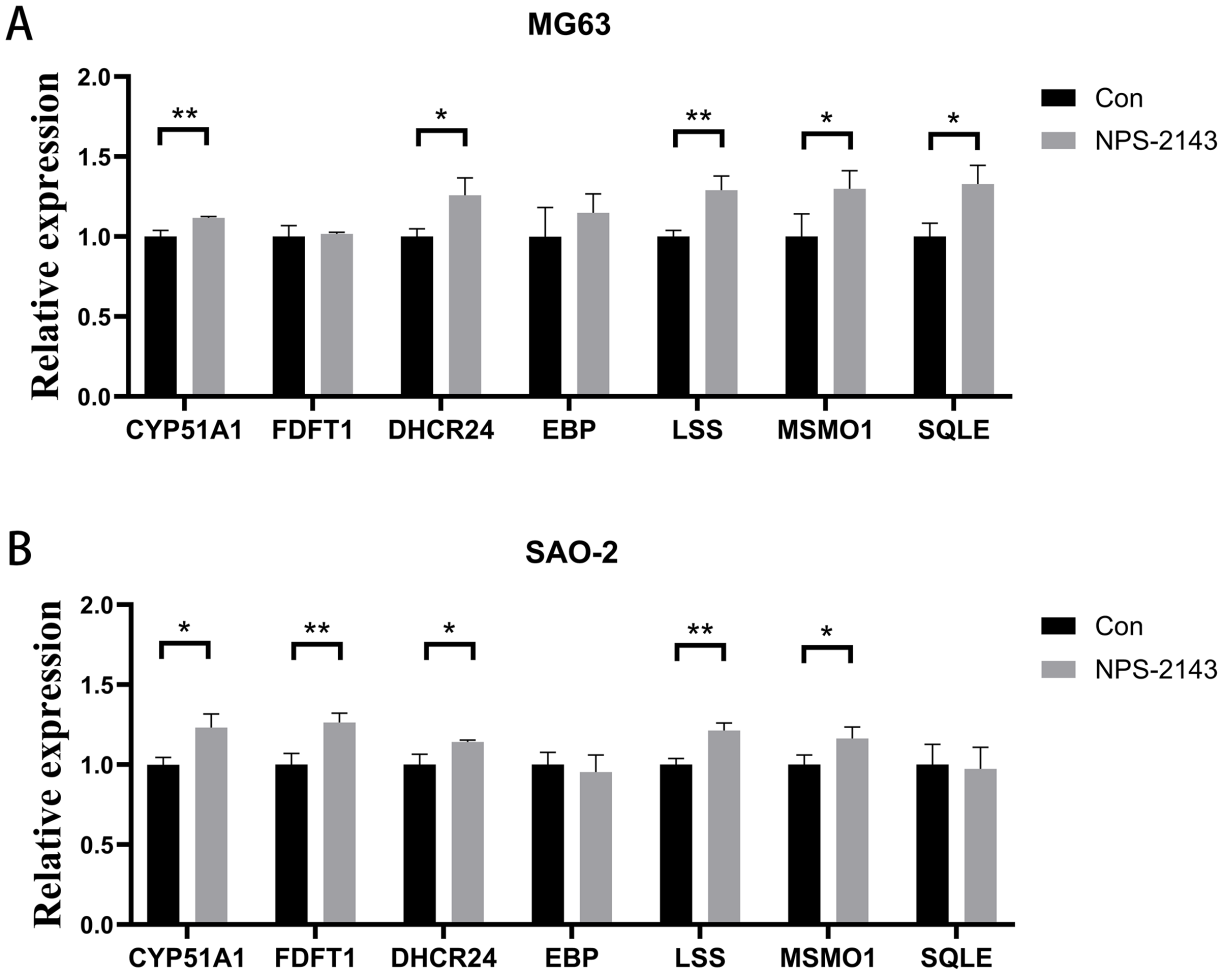
CaSR antagonism promotes expression of genes for cholesterol synthesis and steroid synthesis pathways in human osteosarcoma cells. (A) MG-63; (B) Saos-2. *P < 0.05, ** *P* < 0.01, *** *P* < 0.001.

Seven genes shared by cholesterol synthesis and steroid synthesis pathways were selected for validation by qRT-PCR. Compared with the control group, the NPS-2143-treated MG-63 cell group had increased mRNA levels for *CYP51A1* (*P* = 0.021), *DHCR24* (*P* = 0.017), *LSS* (*P* = 0.005), *MSMO1* (*P* = 0.028), and *SQLE* (*P* = 0.014) (Fig. 4A). The NPS-2143-treated Saos-2 cell group had increased mRNA levels for *CYP51A1* (*P* = 0.022), *DHCR24* (*P* = 0.029), *LSS* (*P* = 0.043), *MSMO1* (*P* = 0.045), and *FDFT1* (*P* = 0.002) (Fig. 4B) compared with the control group, which was consistent with the sequencing results.

## Discussion

CaSR is a G-protein-coupled receptor that is distributed in a wide variety of tissues and is involved in the regulation of Ca^2+^ homeostasis. In recent years, accumulating evidence has shown that CaSR plays an important role in the occurrence and development of various malignant tumors, and that its activation or inhibition may affect the proliferation, apoptosis, invasion, and metastasis of tumor cells (Chakravarti et al., 2009; Ying et al., 2023). In some malignant cancers, such as breast, prostate, and parathyroid cancers, abnormal expression of CaSR is associated with highly aggressive and metastatic tumor cells (Chakravarti et al., 2009; Ying et al., 2023). CaSR is therefore a potential anti-tumor target, and CaSR antagonists, as negative regulators, show promise in regulating tumor cell proliferation and invasion.

The role of CaSR varies according to tumor type, cell microenvironment, and signaling pathway. CaSR can inhibit tumor growth by inhibiting cell proliferation and promoting apoptosis, but it can also promote cell proliferation, invasion and metastasis in some tumors (Brennan et al., 2013). In a variety of malignancies, abnormal activation of CaSR may promote tumor cell survival and invasion. CaSR antagonists can inhibit ERK1/2, PI3K/Akt, and Wnt/β-catenin signaling pathways, thereby reducing the proliferation of tumor cells and promoting apoptosis. Furthermore, during bone metastasis in breast cancer, CaSR promotes the adaptation of cancer cells to the bone microenvironment (Organista-Juarez et al., 2013), while CaSR antagonists may reduce the capacity of tumor cells to invade bone by reducing Ca^2^^+^ dependent signaling. Although CaSR antagonists exhibit antitumor activity in some tumors, in other tumor types CaSR may have an inhibitory effect and the use of CaSR antagonists may instead promote tumor growth (Saidak, Mentaverri & Brown, 2009). While CaSR exhibits divergent roles across cancer types, it has been shown to be upregulated in MG-63 osteosarcoma cells compared to normal osteoblasts *in vitro*, where it may modulate proliferation, apoptosis, and invasion *via* calcium signaling (Zhao et al., 2020). In this study, the MG-63 and Saos-2 cell lines were selected to validate the effects of CaSR antagonism on osteosarcoma cells. Our results confirm that CaSR antagonism inhibits the viability of osteosarcoma cells *in vitro*. Therefore, we suggest that CaSR antagonists have potential for use in the treatment of osteosarcoma.

We analyzed the transcriptomic effects of CaSR antagonism on osteosarcoma cells to identify pathways potentially involved in reduced cell viability. We found that a CaSR antagonist promoted the expression of genes associated with cholesterol and steroid synthesis in osteosarcoma cells. qRT-PCR analysis confirmed that the CaSR antagonist promoted the expression of the cholesterol and steroid synthesis-related genes, *CYP51A1*, *DHCR24*, *LSS*, and *MSMO1* in MG-63 and Saos-2 cells. LSS (Lanosterol synthase) catalyzes one of the earliest steps in sterol biosynthesis by converting 2,3-oxidosqualene to lanosterol, a key intermediate in the production of cholesterol and other sterol compounds (Guo & Zhang, 2023). This process involves a series of complex chemical reactions, such as intramolecular nucleophilic addition, carbocation rearrangement, and proton transfer to form the tetracyclosterane structure and transforming the linear precursor into a steroid skeleton structure (Nes, 2011). The catalytic activity of LSS is essential in the homeostatic regulation of cholesterol and other sterols because lanosterene is the first tetra-closterane compound in the cholesterol synthesis pathway and is further converted into cholesterol and steroid hormones. This enzyme is mainly located in the endoplasmic reticulum, and its abundance and activity are regulated by feedback from the level of cholesterol to maintain the balance of intracellular sterol metabolism. CYP51A1 (Lanosterol 14α-demethylase) functions downstream of LSS in the early-to-mid stage of cholesterol biosynthesis. It is a cytochrome P450 enzyme that catalyzes the conversion of lanosterol to 4,4-dimethylcholesta-8(9),14,24-trien-3β-ol (Akhtar & Wright, 2015). Cytochrome P450 enzymes are a conserved protein family involved in the metabolism of organic compounds and the biosynthesis of steroids, lipids, and vitamins in eukaryotes. As part of this family, lanosterol 14α-demethylase plays a critical role in sterol biosynthesis by removing the C-14α-methyl group from lanosterol. This demethylation process is the initial control point for the biosynthesis of cholesterol and other sterols, and is critical for ensuring sterol hormone synthesis (Lepesheva & Waterman, 2007). MSMO1 (methylsterol monooxygenase 1) acts at the mid-stage of cholesterol biosynthesis and serves as a key enzyme in this pathway. It primarily catalyzes the three-step monooxygenation required for the demethylation of 4,4-dimethyl and 4alpha-methylsterols, which can be subsequently metabolized to cholesterol. This process is essential for subsequent cholesterol synthesis steps because the correct structure of cholesterol requires the removal of excess methyl groups (He et al., 2011a; Mazein et al., 2013). DHCR24 (δ24-cholesterol reductase, also known as 3β-hydroxysterol Δ24-reductase) is an enzyme that catalyzes the final step in the cholesterol biosynthesis pathway (mevalonate pathway), converting desmosterol to cholesterol (Bai et al., 2022; Fu & Wang, 2024; Zerenturk et al., 2013). Collectively, these four enzymes—LSS, CYP51A1, MSMO1, and DHCR24—cover sequential reactions from the early to the terminal stages of the cholesterol biosynthetic pathway, offering an integrated view of sterol metabolism and suggesting that CaSR signaling may intersect with multiple levels of cholesterol regulation in osteosarcoma cells. Cholesterol synthesis plays a key role in maintaining cell membrane stability, signal transduction, and cell metabolism. Accumulating evidence shows that disrupted cholesterol metabolism is closely related to the occurrence, development, and drug resistance of various malignant tumors. Tumor cells have a high demand for cholesterol to maintain the membrane lipid bilayer stability required for rapid proliferation and survival. At the same time, cholesterol is also a precursor of various steroid hormones and signaling molecules, which play an important role in cancer progression (Huang, Song & Xu, 2020). Inhibitors that target cholesterol synthesis, such as HMG-CoA reductase inhibitors (statins), have anti-tumor effects in multiple cancer types (Tutuska et al., 2020). While tumor cells can drive metabolically dependent proliferation by abnormally activating cholesterol synthesis pathways, our findings suggest that CaSR antagonism may modulate cholesterol biosynthesis-related pathways at the transcriptomic level.

Although cholesterol is essential for maintaining cell membrane integrity, signal transduction, and cell metabolism, its excessive production can disrupt cellular homeostasis. Cholesterol biosynthesis involves multiple redox processes, and excessive cholesterol synthesis leads to the accumulation of reactive oxygen species, which in turn triggers oxidative stress (Guo et al., 2024). Excessive cholesterol synthesis can also lead to mitochondrial dysfunction, DNA damage, protein oxidation, and lipid peroxidation, which ultimately affect cell proliferation and promote apoptosis (Liu, 2024; Xiao et al., 2023). In addition, excess intracellular cholesterol can alter cell membrane composition, increase membrane rigidity and destroy lipid rafts, inhibit key pathways such as PI3K/Akt/mTOR and MAPK/ERK, reduce cell reactivity to growth signals, and limit proliferation (Xiao et al., 2023). Excess cholesterol can be oxidized to form oxysterols, which can activate a variety of signaling pathways leading to the production of pro-inflammatory cytokines. Cholesterol overload also reduces autophagy activity, leading to cell dysfunction. Autophagy is a cellular process in which damaged organelles and proteins are degraded and recycled to maintain cell homeostasis. Accumulation of too much cholesterol impairs autophagy, leading to a build-up of cell debris and organelle dysfunction. This damage can reduce cell proliferation and trigger cell death pathways (Guo et al., 2024; Xiao et al., 2023). These results suggest that excessive cholesterol synthesis may contribute to the activation of cell death pathways and the suppression of cell viability. This finding provides preliminary evidence that CaSR antagonists could exert anti-tumor effects by modulating cholesterol biosynthesis, offering a potential mechanistic link warranting further investigation. This study has several limitations. All experiments were conducted *in vitro* using osteosarcoma cell lines, which may not fully reflect the complexity of tumor behavior *in vivo*. Additionally, the absence of *in vivo* or clinical validation restricts the translational relevance of our findings. Further studies involving animal models and patient-derived samples are warranted to verify the mechanistic insights and assess the therapeutic potential of CaSR antagonists in osteosarcoma. Another limitation is the lack of non-malignant control cells, which precludes evaluation of the compound’s selectivity for tumor *versus* normal cells. Future work will incorporate normal osteoblasts or mesenchymal stem cells to examine the effects of NPS-2143 on non-tumor cell viability.

## Conclusions

This study demonstrates that CaSR antagonism reduces the viability of human osteosarcoma cells *in vitro*. Transcriptomic analysis revealed upregulation of key genes involved in cholesterol and steroid biosynthesis following treatment with NPS-2143. These findings suggest that targeting CaSR may disrupt lipid metabolism and represent a potential therapeutic strategy for osteosarcoma. Further *in vivo* studies are needed to validate these results and explore their translational relevance.

##  Supplemental Information

10.7717/peerj.20546/supp-1 Supplemental Information 1 CCK8

10.7717/peerj.20546/supp-2 Supplemental Information 2 MG63 KEGG enrichment analysis(MNKPA001FC159)

10.7717/peerj.20546/supp-3 Supplemental Information 3 MG63 Reactome enrichment analysis(MNKPA001FC159)

10.7717/peerj.20546/supp-4 Supplemental Information 4 SAO-2 KEGG enrichment analysis(SNKPA001FC1927)

10.7717/peerj.20546/supp-5 Supplemental Information 5 SAO-2 Reactome enrichment analysis(SNKPA001FC1927)

10.7717/peerj.20546/supp-6 Supplemental Information 6 SAO-2 and MG63 KEGG enrichment analysis(SUMNKPA001FC127)

10.7717/peerj.20546/supp-7 Supplemental Information 7 SAO-2 and MG63 Reactome enrichment analysis(SUMNKPA001FC127)

10.7717/peerj.20546/supp-8 Supplemental Information 8 PCR MG63

10.7717/peerj.20546/supp-9 Supplemental Information 9 PCR SAO-2

10.7717/peerj.20546/supp-10 Supplemental Information 10 MIQE Checklist

10.7717/peerj.20546/supp-11 Supplemental Information 11 Supplementary Materials

10.7717/peerj.20546/supp-12 Supplemental Information 12 Primer Sequences and Amplification Efficiencies Used for qRT-PCR

10.7717/peerj.20546/supp-13 Supplemental Information 13 Bioinformatic Analysis

## Additional Information and Declarations

### Data Availability

The following information was supplied regarding data availability: The raw measurements are available in the Supplementary Files.
