# Supplementary material for: Cholesterol and steroid synthesis pathways may be involved in the inhibition of osteosarcoma cell viability by calcium-sensing receptor antagonism
Source: PeerJ. 2026 Jan 5;14:e20546. doi: 10.7717/peerj.20546 (PMC12782028; doi:10.7717/peerj.20546)
Supplement: Supplemental Information 11 [file peerj-14-20546-s011.docx]

**EXPERIMENTAL DESIGN**

**Definition of experimental and control groups**

Sao-2 cells were divided into two groups: control, NPS-2143.; Additionally, MG63 cells were divided into two groups: control, NPS-2143. To investigate the impact of NPS-2143 on human osteosarcoma cells, the cells in the NPS-2143 group were treated with 5 µM NPS-2143 for 24 hours, following established protocols. After another 24-hour culture period, the cells were collected for subsequent experiments.

Number within each group

The biological replicates were 3 for each group.

**SAMPLE**

**Description**

Sao-2 and MG63 cells were inoculated with a density of 2×10^5^/ml and cultured with 6-well plates, and RNA was extracted after experimental intervention.

**Processing procedure**

If frozen - how and how quickly?

No frozen.

If fixed - with what, how quickly?

No fixed.

Sample storage conditions and duration (especially for FFPE samples)

Total RNA was extracted by TRIzol method immediately after the intervention.

**NUCLEIC ACID EXTRACTION**

**Procedure and/or instrumentation**

**Name of kit and details of any modifications**

RNA was obtained from Sao-2 and MG63 samples using TRIzol® reagent. Specific operations are as follows

​ After the culture solution was sucked out, 1ml of Trizol was added to each hole of the 6-well culture plate, and the solution of Trizol cells was blown up and sucked into a 1.5ml dynamic EP tube.

Absorb Trizol into 1.5ml dynamic EP tube (apply dynamic gun tip)

Let stand at room temperature for 5 minutes;

Add 0.3ml of chloroform, cover tightly and mix manually for 15 seconds;

2 - 3 minutes for the temperature;

Low temperature centrifuge 4℃, 12000g centrifuge for 15 minutes;

Approximately 0.4ml of the supernatant is transferred to a 1.5ml dynamic EP tube;

Add 0.5ml of isopropyl alcohol, mix well and let stand at room temperature for 10 minutes;

Low temperature centrifuges, 10,000 g centrifuges for 10 minutes;

Discard the supernatant, add 1ml 75% ethanol (prepared with DEPC treated water) and mix well;

Low temperature centrifuge 4℃, 7000g centrifuge for 5 minutes;

Discard the supernatant and dry at room temperature for 5 minutes;

DEPC treated water is dissolved in 20ul and stored at -80℃

The total RNA concentration of the sample was detected using an ultraviolet spectrophotometer;

**Details of DNase or RNAse treatment**

RNA was obtained from Sao-2 and MG63 samples using TRIzol® reagent and reverse-transcribed with the PrimeScript™ RT reagent Kit with gDNA Eraser (Perfect Real Time RR047A) (Takara). All experimental procedures were performed as specified in the kit instructions. The kit contains the gDNA Eraser, which removes genomic DNA in exactly 2 minutes. Among them, the reagent PrimeScript RT Enzyme Mix I has an RNase inhibitor.

**Contamination assessment (DNA or RNA)**

OD_260/280_ values of RNA samples were detected by ultraviolet spectrophotometer (Thermo), and OD_260/280_ values of all samples were greater than 1.8 and less than 2.

**Nucleic acid quantification**

**Instrument and method**

The RNA to be measured was dropped into the detection area, and the RNA concentration of the sample was detected by ultraviolet spectrophotometer.

**RNA integrity method/instrument**

RNA integrity was monitored by agarose gel electrophoresis, and the 28S/18S of each sample were between 1.7 and 2.1.

**Inhibition testing (Cq dilutions, spike or other)**

The target gene dissolution curves of all samples were unimodal.

**REVERSE TRANSCRIPTION**

**Complete reaction conditions**

**Amount of RNA and reaction volume**

Step 1

5×gDNA Eraser Buffer 2.0 μl

gDNA Eraser 1.0 μl

Total RNA 1.0 ng

RNase Free dH2O up to 10 μl

42℃ 2 min

4℃

Step 2

The reaction liquid of Step 1 10.0 μl

PrimeScript RT Enzyme Mix I 1.0 μl

RT Primer Mix 1.0 μl

5×PrimeScript Buffer 2 4.0 μl

RNase Free dH2O 4.0 μl

Total 20 μl

37℃ 15 min

85℃ 5 sec

4℃

**Priming oligonucleotide (if using GSP) and concentration**

RNA was reverse-transcribed with the PrimeScript™ RT reagent Kit with gDNA Eraser (Perfect Real Time RR047A) (Takara) in this experiment. The kit includes reagent PrimeScript Buffer, which contains dNTP Mixture.

**Reverse transcriptase and concentration**

RNA was reverse-transcribed with the PrimeScript™ RT reagent Kit with gDNA Eraser (Perfect Real Time RR047A) (Takara). The kit includes reagent PrimeScript RT Enzyme Mix I, which contains Reverse transcriptase and RNase Inhibitor.

**Temperature and time**

37℃ 15 min

85℃ 5 sec

4℃

**Manufacturer of reagents and catalogue numbers**

PrimeScript™ RT reagent Kit with gDNA Eraser (Perfect Real Time RR047A) (Takara)

**qPCR TARGET INFORMATION、PROTOCOL 、OLIGONUCLEOTIDES and VALIDATION**

The primer sequence used in this study is from <https://pga.mgh.harvard.edu/> primerbank/, which has been validated and used. TB Green Premix Ex Taq II（Tli RNaseH Plus）(Code No. RR820A) was selected for this study. All experimental procedures were conducted as specified in the kit instructions.

**Probe sequence**

**SQLE**

Gene Descriptions:

NCBI GeneID 6713

GenBank Accession NM_003129

NCBI Protein Accession NP_003120

Species Human

Coding DNA Length 1725

Gene Description Homo sapiens squalene epoxidase (SQLE), mRNA.

PrimerBank ID 62865634c1

Amplicon Size 202

Sequence (5' -> 3') Length Tm Location

Forward Primer GGCATTGCCACTTTCACCTAT 21 60.6 16-36

Reverse Primer GGCCTGAGAGAATATCCGAGAAG 23 61.6 217-195

**LSS**

NCBI GeneID 4047

GenBank Accession NM_001001438

NCBI Protein Accession NP_001001438

Species Human

Coding DNA Length 2199

Gene Description Homo sapiens lanosterol synthase (2,3-oxidosqualene-lanosterol cyclase) (LSS), transcript variant 2, mRNA.

PrimerBank ID 221139912c1

Amplicon Size 104

Sequence (5' -> 3') Length Tm Location

Forward Primer GCACTGGACGGGTGATTATGG 21 62.8 276-296

Reverse Primer TCTCTTCTCTGTATCCGGCTG 21 60.4 379-359

**EBP**

NCBI GeneID 10682

GenBank Accession NM_006579

NCBI Protein Accession NP_006570

Species Human

Coding DNA Length 693

Gene Description Homo sapiens emopamil binding protein (sterol isomerase) (EBP), mRNA.

PrimerBank ID 169808388c1

Amplicon Size 94

Sequence (5' -> 3') Length Tm Location

Forward Primer CTCAGCACCTAAGACTGGACA 21 60.9 38-58

Reverse Primer ACGACTAAGACCCCTGTGACA 21 62.3 131-111

**DHCR24**

NCBI GeneID 1718

GenBank Accession NM_014762

NCBI Protein Accession NP_055577

Species Human

Coding DNA Length 1551

Gene Description Homo sapiens 24-dehydrocholesterol reductase (DHCR24), mRNA.

PrimerBank ID 114155130c1

Amplicon Size 144

Sequence (5' -> 3') Length Tm Location

Forward Primer GCCGCTCTCGCTTATCTTCG 20 63.0 120-139

Reverse Primer GTCTTGCTACCCTGCTCCTT 20 61.3 263-244

**CYP51A1**

NCBI GeneID 1595

GenBank Accession NM_000786

NCBI Protein Accession NP_000777

Species Human

Coding DNA Length 1530

Gene Description Homo sapiens cytochrome P450, family 51, subfamily A, polypeptide 1 (CYP51A1), transcript variant 1, mRNA.

PrimerBank ID 168693652c1

Amplicon Size 76

Sequence (5' -> 3') Length Tm Location

Forward Primer GAAACGCAGACAGTCTCAAGA 21 60.0 825-845

Reverse Primer ACGCCCATCCTTGTATGTAGC 21 61.9 900-880

**FDFT1**

NCBI GeneID 2222

GenBank Accession NM_004462

NCBI Protein Accession NP_004453

Species Human

Coding DNA Length 1254

Gene Description Homo sapiens farnesyl-diphosphate farnesyltransferase 1 (FDFT1), mRNA.

PrimerBank ID 67089146c1

Amplicon Size 134

Sequence (5' -> 3') Length Tm Location

Forward Primer CCACCCCGAAGAGTTCTACAA 21 61.1 24-44

Reverse Primer TGCGACTGGTCTGATTGAGATA 22 60.6 157-136

**MSMO1**

NCBI GeneID 6307

GenBank Accession NM_001017369

NCBI Protein Accession NP_001017369

Species Human

Coding DNA Length 489

Gene Description Homo sapiens methylsterol monooxygenase 1 (MSMO1), transcript variant 2, mRNA.

PrimerBank ID 345525411c1

Amplicon Size 143

Sequence (5' -> 3') Length Tm Location

Forward Primer TGCTTTGGTTGTGCAGTCATT 21 61.0 31-51

Reverse Primer GGATGTGCATATTCAGCTTCCA 22 60.4 173-152

**DATA ANALYSIS**

**qPCR analysis program (source, version)**

Roche Group LightCycler96.

**Cq method determination**

Cq method=15.

**Outlier identification and disposition**

No outliers appear in this experiment.

**Results of NTCs**

No specific peak of NTC appeared.

**Justification of number and choice of reference genes**

The relative expression was calculated using the 2^−△△Ct^ method and estimated relative to GAPDH.

**Description of normalisation method**

All data were normalized by dividing by the mean of the control group before statistical analysis.

**Number and stage (RT or qPCR) of technical replicates**

​During the experimental sample addition process, each sample was repeated with 2 Wells.

**Repeatability (intra-assay variation)**

In this experiment, the difference of CT values between the two holes was less than 0.1.

**Statistical methods for result significance**

For qRT-PCR analysis, the differences among the three groups were evaluated using a one-way analysis of variance (ANOVA) followed by a post hoc test to determine the least significant difference. A significance level of P < 0.05 was considered statistically significant.

**Software (source, version)**

IBM SPSS Statistics 29
