# Supplementary material for: Cholesterol and steroid synthesis pathways may be involved in the inhibition of osteosarcoma cell viability by calcium-sensing receptor antagonism
Source: PeerJ. 2026 Jan 5;14:e20546. doi: 10.7717/peerj.20546 (PMC12782028; doi:10.7717/peerj.20546)
Supplement: Supplemental Information 13 [file peerj-14-20546-s013.docx]

**Bioinformatic Analysis**

**1. Raw Sequence Data Quality Assessment**

High-throughput sequencing was performed using a second-generation sequencing platform. Statistical methods were applied to evaluate the base composition and quality fluctuations across all sequencing cycles for each dataset. Quality control of the raw sequencing reads was conducted for each sample, including:

1. analysis of A/T/G/C base composition distribution;
2. assessment of base quality score distribution; and
3. calculation of base error rate distribution.

**2. Quality Control of Raw Sequencing Data**

To ensure the accuracy of subsequent bioinformatic analyses, raw sequencing reads were first filtered to obtain high-quality clean data for downstream processing. The specific steps were as follows:

1. adapter sequences and reads without insert fragments (caused by self-ligation of adapters) were removed;
2. low-quality bases (Phred score < 20) at the 3′ end of reads were trimmed, and reads were discarded if any remaining base had a quality score < 10;
3. reads containing more than 10% ambiguous bases (N) were removed; and
4. reads shorter than 20 bp after adapter trimming and quality filtering were discarded.

After quality control, the clean reads were re-evaluated by calculating the base error rate and base composition distributions to confirm data quality.

All filtering and quality control procedures were performed using Fastp (https://github. com/OpenGene/fastp).

**3. Alignment to the Reference Genome**

The high-quality clean reads obtained after quality control were aligned to the reference genome to generate mapped data for downstream transcript assembly and expression quantification. The alignment results from this RNA-seq analysis were further evaluated for quality, including sequencing saturation, gene coverage, read distribution across different genomic regions, and read distribution among chromosomes.

All alignments were performed using HISAT2 (http://ccb.jhu.edu/software/hisat2/index.shtml).

**4. Expression Quantification**

In RNA-Seq analysis, gene expression levels were quantified based on the number of clean reads mapped to the corresponding genomic regions (read counts). The software RSEM was used to estimate expression levels at both the gene and transcript levels, providing quantitative data for subsequent differential expression analyses among samples. By integrating functional annotation, these data also facilitated the exploration of gene regulatory mechanisms.

Software used: RSEM (http://deweylab.github.io/RSEM/)

**5. Differential Expression Analysis**

After obtaining the read count values for each gene, differential expression analysis was performed to identify genes that were significantly differentially expressed between samples. The software DESeq2 was used to conduct statistical comparisons of gene expression levels across groups. Genes with a false discovery rate (FDR) < 0.01 and an absolute fold change (|FC|) ≥ 1 were defined as differentially expressed genes (DEGs).

Software used: DESeq2 (<http://bioconductor.org/packages/stats/bioc/DESeq2/>)

**6. Reactome and KEGG Annotation of Differentially Expressed Genes**

The Reactome and KEGG databases were used to categorize differentially expressed genes (DEGs) according to their associated biological pathways and functional classifications. Functional annotation of DEGs was performed based on their participation in specific pathways identified in these databases.

Databases used: Reactome (https://reactome.org/) and KEGG (<https://www.genome>. jp/kegg/)

**7. Reactome and KEGG Enrichment Analysis of Differentially Expressed Genes**

Enrichment analysis of differentially expressed genes (DEGs) was performed using the Python SciPy package. The Fisher’s exact test was applied to assess pathway enrichment, and the Benjamini–Hochberg (FDR) method was used to correct for multiple testing. Pathways with a corrected p-value (adjusted p-value) < 0.05 were considered significantly enriched among the DEGs.

Software used: Python SciPy package (<https://scipy.org/install/>)
